# Animal social networks are robust to changing association definitions

**DOI:** 10.1007/s00265-025-03559-7

**Published:** 2025-02-06

**Authors:** Alex Hoi Hang Chan, Jamie Dunning, Kristina B Beck, Terry Burke, Heung Ying Janet Chik, Daniel Dunleavy, Tim Evans, André Ferreira, Babette Fourie, Simon C. Griffith, Friederike Hillemann, Julia Schroeder

**Affiliations:** 1 Centre for the Advanced Study of Collective Behaviour, University of Konstanz, Konstanz, Germany; 2 Department of Collective Behaviour, Max Planck Institute of Animal Behaviour, Konstanz, Germany; 3 Department of Life Sciences, Imperial College London, Silwood park, Ascot, UK; 4 Faculty of Biological Sciences, University of Leeds, Leeds, UK; 5 Department of Biology, Edward Grey Institute, University of Oxford, Oxford, UK; 6 Ecology and Evolutionary Biology, School of Biosciences, The University of Sheffield, Sheffield, UK; 7 Groningen Institute for Evolutionary Life Sciences, University of Groningen, Groningen, Netherlands; 8 School of Natural Sciences, Macquarie University, Sydney, Australia; 9 Center for Complexity Science, Imperial College London, London, UK; 10 Centre d’Ecologie Fonctionnelle et Evolutive, University Montpellier, Montpellier, France; 11 Centro de Investigação em Biodiversidade e Recursos Genéticos, University of Porto, Porto, Portugal; 12 Max Planck Institute for Evolutionary Anthropology, Leipzig, Germany

**Keywords:** Animal behaviour, Gambit of the group, Sociality, Social network, RFID

## Abstract

The interconnecting links between individuals in an animal social network are often defined by discrete, directed behaviours, but where these are difficult to observe, a network link (edge) may instead be defined by individuals sharing a space at the same time, which can then be used to infer a social association. The method by which these associations are defined should be informed by the biological significance of edges, and therefore often vary between studies. Identifying an appropriate measure of association remains a challenge to behavioural ecologists. Here, we use automatically recorded feeder visit data from four bird systems to compare three methods to identify a social association: (1) strict time-window, (2) co-occurrence in a group, and (3) arrival-time. We tested the similarity of the resulting networks by comparing the repeatability and sensitivity of individuals’ social traits (network degree, strength, betweenness). We found that networks constructed using different methods but applying similar, ecologically relevant definitions of associations based on individuals’ spatio-temporal co-occurrence, showed similar characteristics. Our findings suggest that the different methods to construct animal social networks are comparable, but result in subtle differences driven by species biology and feeder design. We urge researchers to carefully evaluate the ecological context of their study systems when making methodological decisions. Specifically, researchers in ecology and evolution should carefully consider the biological relevance of an edge in animal social networks, and the implications of adopting different definitions.

**Supplementary Information:**

The online version contains supplementary material available at 10.1007/s00265-025-03559-7.

## Significance Statement

Time series data containing animal identities in physical and temporal proximity are commonly used to infer social networks from wild animals. To identify biologically-relevant social associations, researchers develop definitions specific to their study system. However, researchers’ degrees of freedom in defining what constitutes a social association (as opposed to discrete interactions, which are usually well-defined) and the implications of using different definitions are often ignored. Here, we compare the structure of networks generated using three methods of social association definitions applied to spatio-temporal data streams from four avian study systems. We found that networks are largely robust to changing social association definitions, with subtle differences. Our results underpin the importance of a priori association definition to infer social networks, as small decisions can influence structures of networks. Researchers should provide validation or biological justification when defining social network edges based on association in time and space.

**Supplementary Information:**

The online version contains supplementary material available at 10.1007/s00265-025-03559-7.

## Introduction

Sociality - one’s propensity to socialise with others - has important consequences for life history and evolutionary processes (Wey et al. 2008; Maldonado-Chaparro et al. 2018). For example, sociable individuals can experience increased survival or reproductive success (Silk et al. 2009; Oh and Badyaev 2010; Formica et al. 2012; Stanton and Mann 2012; Maldonado-Chaparro et al. 2018; Dunning et al. 2023) and better access to information and resources (e.g., Aplin et al. 2012), compared to less sociable individuals. The social structure of a population also has implications for the transmission of infectious disease and information (Keeling and Eames 2005; McDonald et al. 2013; Kurvers et al. 2014; Aplin et al. 2015b; Albery et al. 2021; Beck et al. 2023). Such social dynamics are generally measured using social networks, defined by a set of nodes and interconnecting edges. Social network analysis is commonly used to quantify connections between individuals (Croft et al. 2008; Wey et al. 2008) and the importance of social links has been demonstrated across systems (in birds: Covas et al. 2006; McDonald 2007; Oh and Badyaev 2010; Firth et al. 2016; in mammals: reviewed in Silk 2007; and in invertebrates; Formica et al. 2012; Wice and Saltz 2021; Cook et al. 2023; and, among individuals of different species: Hillemann et al. 2019). However, the definitions of these links - what constitutes a social relationship - and the methods used to define them vary among studies.

In the wild, it can be challenging to directly observe social interactions among individuals and so, social connectivity is often inferred from individuals overlapping in space and time – an *aggregation*. *Aggregations*, while generally describing spatio-temporal co-occurrence, may vary in their intentionality, intensity and duration. For example, moths gathering around a light source can be explained by proximate causes rather than by social or intentional association (Tinbergen 1953). Researchers often then seek to extract social data from aggregations of animals by defining parameters to extract something proximal to social intentionality, that is, that two or more individuals choose to associate with each other, over all others. We refer to this as an *association.* We refer to groupings, driven by non-social factors, as *aggregations* (following Krause and Ruxton 2002).

The need to distinguish between different forms of aggregation is dependent on research objectives. For example, non-social aggregation may be more relevant than the strength and identity of social associations in diluting predation risk or behavioural transmission (for example Cresswell 1994; Krause and Ruxton 2002; Sorato et al. 2012; Voelkl et al. 2016, Firth 2020); Whereas. the identity and behavioural preferences of associating individuals is important when measuring the drivers of individual mate choice (Oh and Badyaev 2010; Wascher et al. 2015; Beck et al. 2021; Dunning et al. 2023). The question then, of how social associations are extracted from aggregations of individual animals is long-standing in animal behaviour studies, and open to interpretation.

Recent methodological advances, from Radio-frequency identification (RFID) feeder design (Bridge et al. 2019; Youngblood 2019), data processing (Farine 2017; Iserbyt et al. 2018), and hypothesis testing (Hart et al. 2022a, b), aim to discern social associations through membership of discrete social groups – the gambit of the group method (Whitehead and Dufault 1999; Franks et al. 2010). This gambit of the group method assumes that individuals that overlap in space and time, to some extent, are associated with each other, hence co-occurring individuals are treated as a social group. Such associations can be inferred from temporal presence/absence data at central resources. These approaches are typically used for species that are difficult to observe in the wild, like small mammals (Godsall et al. 2014; Evans et al. 2021; Raulo et al. 2021) or birds, that feed and shelter communally (Ringsby et al. 2009; Mariette et al. 2011; Farine 2017; nchez-Tójar et al. 2017; Bandivadekar et al. 2018; Firth et al. 2018; Broughton et al. 2019; Hillemann et al. 2020), but defining what constitutes a social unit presents a challenge.

**Fig. 1:**
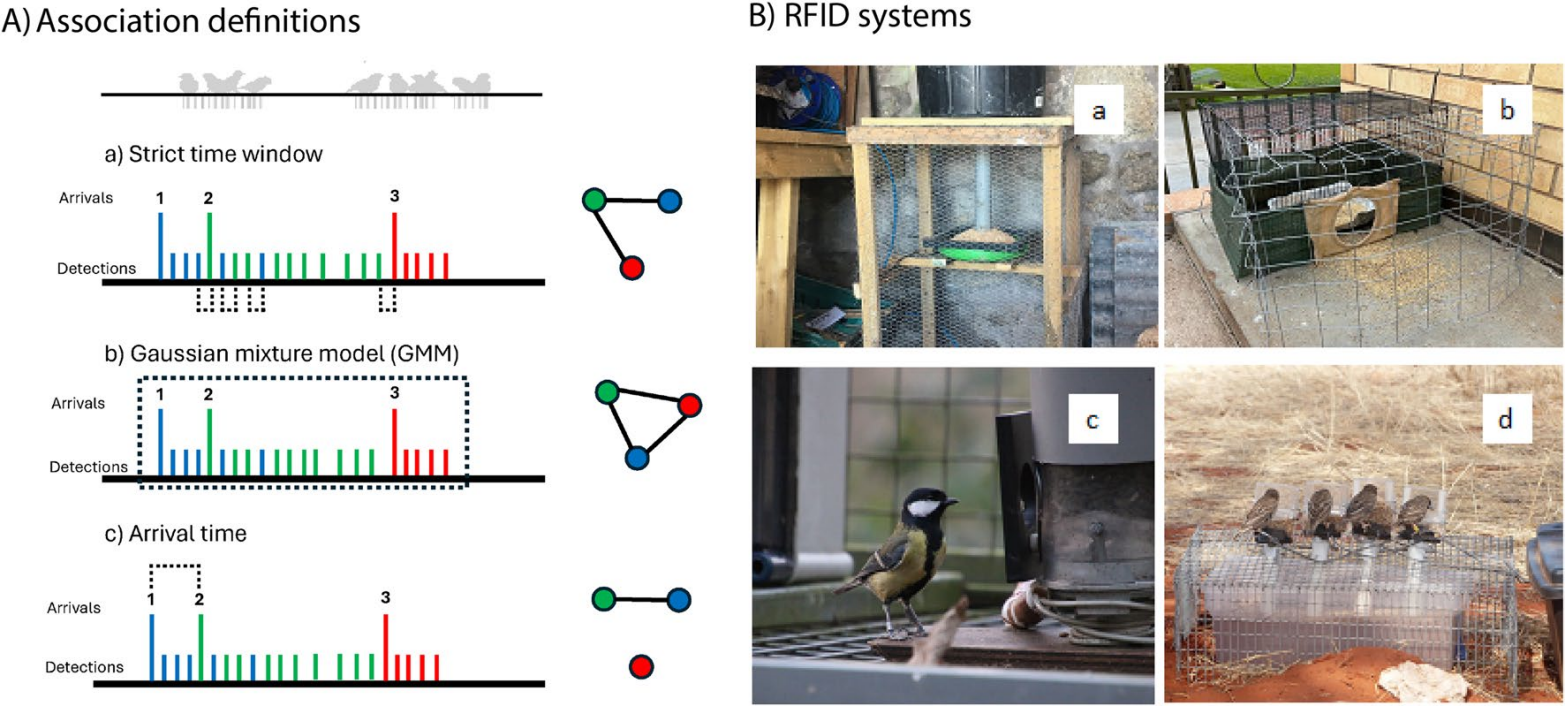
** A** Three association definitions applied to sampled RFID data streams. Black lines denote individuals visiting a feeder where three are highlighted (one, two and three). The time-window definition (Aa) where individuals are considered associating when they overlap in space within (Δ t) of each other. The GMM (Ab) definition identifies the start and the end of gathering events, denoted here with red dotted lines. Finally, arrival-time (Ac) defines an association where individuals overlap by Δ t of their arrival (first detection) at the RFID antenna. An individual can only be recorded as arriving again after a period of absence defined by Δ i. **B**. Radio Frequency Identification (RFID) experimental set-up in four systems: (**a**) open RFID antenna on Lundy Island, UK house sparrows Passer domesticus ; (**b**) RFID antenna at the entrance to a feeding chamber at Broken Hill, Australia for house sparrows; (**c**) Great tit Parus major interacting with a single RFID antenna at Wytham woods, UK; (**d**) Sociable weaver Philetairus socius visiting four RFID antenna mounted at Benfontein Nature Reserve, South Africa

One common approach to infer associations from these data, is to use a *strict time-window* (**Δ**t; Fig. 1Aa) within which all individuals co-occurring at the same location and time are defined as socially associating. For example, associations have been defined between PIT (Passive Integrated Transponder)-tagged house sparrow *Passer domesticus*, foraging at the same RFID feeder within three seconds of each other (Plaza et al. 2019). Where spatial and temporal proximity is most important for the research question, a time window approach is the simplest method for defining spatio-temporal overlap. However, if the time-window is too short, socially associated individuals may not be identified as belonging to the same group, yet, if the time-window is too long, associations are inappropriately defined within non-social aggregations of birds (Croft et al. 2008; Psorakis et al. 2015).

To solve this problem, gaussian mixture models have been developed to identify discrete grouping events in temporal data streams (GMM; Psorakis et al. 2012, 2015; Fig. 1Ab). The GMM approach considers dynamically changing time-windows, determined by periods of increased activity at a bird feeder (Psorakis et al. 2012). The publication of an associated R package “asnipe” (Farine 2013) has led to popular usage across many bird (see, Moyers et al. 2018; Broughton et al. 2019; Evans and Morand-Ferron 2019; Whiteside et al. 2019; Taff et al. 2019; Brandl et al. 2021; Madsen et al. 2021; Beltrão et al. 2022), and non-bird systems (Findlay et al. 2016; Poirier and Festa-Bianchet 2018; Zeus et al. 2018; Skinner and Miller 2020).

Similarly to the strict time-window, the biological validity of using a GMM approach depends on the biology of the species and and experimental design study system. The GMM approach was specifically designed to identify bursts of activity at bird feeders, and was developed for a system of foraging great tits *Parus major*, which form small fission-fusion flocks over the non-breeding season. This approach may not be suitable for other systems; for example, house sparrow, that form highly gregarious flocks with loose group-level social preferences (Tóth et al. 2009; Havlíček et al. 2022; Dunning et al. 2023). In such gregarious systems, GMMs may struggle to identify group limits, preventing the definition of associations within aggregations of birds at a feeder. This can be problematic if the researchers are more interested in questions where the identities of socially associated individuals matter.

In systems like house sparrows, the time between the arrival of individuals to a feeder may be more suitable to infer associations (hereafter Arrival-time approach; Dunning et al. 2023; Chan and Dunning 2023; Fig. 1Ac). Unlike the strict time window approach and GMM, the arrival-time approach assumes that flocks of socially associated individuals are more likely to arrive together at a resource, than to individuals who are not, capturing finer-scale movement behaviours between associated individuals rather than proximity at a feeder (McKinnon et al. 2006; Atton et al. 2012; Hilleman et al. 2020).

Determining an appropriate definition for the edges in a social network, such that they represent a social association, is not trivial; while decisions are based on the scientific questions of interest and should consider the study system’s biology, these a priori decisions (Castles et al. 2014; Carter et al. 2015; Farine 2015; Farine and Whitehead 2015), and often applied without biological justification or validation steps. Yet, there is limited appraisal in the literature on how (1) different association definitions can influence the resulting social network structure, when applied to the same data stream; and, (2) how the same methods compares across different study systems of different species and with subtly different social behaviours.

Here, we test how different methodological approaches used to infer associations in animal social behaviour studies can influence resulting social networks in systems with different biology and behaviours. To this end, we tested the effect of three commonly used methods (1. strict time-window, 2.GMM, 3. arrival time), in four avian study systems, and described the factors that influence the resulting network topology, individual network position and its repeatability. Additionally, we compared the performance of the approaches under different parameterisation (e.g., varying the time window from 1 to 300 s for the strict time-window and arrival time approaches). With the prevalent use of temporal data streams to infer social networks in behavioural ecology, our results can inform animal behaviour researchers on the consequences of their methodological choices when working with spatio-temporal data.

## Materials and methods

### Systems

We collected data from wild PIT-tagged birds at four systems: Two house sparrow populations, at Broken Hill, Australia, and Lundy Island, UK; one sociable weaver population at Benfontein Nature reserve, South Africa; and a great tit population at Wytham woods, UK. In all four systems, RFID antennas were mounted at a bird feeder to record PIT tagged individuals (Fig. 1B). All data collection was automated, and blind methods were used when all behavioral data were analyzed.

1. House sparrow on Lundy Island, Devon, UK (51.11 N, 4.40 W): We collected RFID data between November 2015 and January 2016 from the long-term study of house sparrows on Lundy Island where they breed and winter around a small village at the island’s southern end. House sparrows are socially monogamous and territorial during the breeding season, nesting in cavities, often in loose colonies. During the non-breeding period house sparrows form gregarious flocks, and often aggregate around food sources (Havlíček et al. 2022). On Lundy, we caught > 95% of the house sparrow population (Simons et al 2015) and fitted subcutaneous PIT tags, which had no effect on fitness (Schroeder et al. 2011). Individuals were recorded visiting a single, 7.8” x 7.8” RFID antenna (DorsetID, Netherlands) mounted below a large seed reservoir and positioned centrally in the study system (Fig. 1Ba), within the home range of all sparrows in the population. Several sparrows can access the feeder at the same time. PIT tags were read approximately every 0.25 s. The RFID system was active when power was available between 6:00 and 00:35 daily (Schroeder et al. 2012).
2. House sparrow at Broken Hill, New South Wales, Australia (31.57 S, 141.27E): We used data from house sparrows visiting one RFID feeding station throughout the non-breeding period in July and August 2022, in Broken Hill, a town of around 1,900 hectares. The ecology of the house sparrows here is broadly like those on Lundy Island. A single feeding station had an RFID antenna fitted around an entrance hole (Priority 1 RFID, Melbourne, Australia) that led to a feeding chamber, inside a metal net cage (Fig. 1Bb). This feeder was in the home-range of only a small proportion of the Broken Hill sparrow population of approximately 19,000 individuals, of which less than ~ 2% were tagged. Sparrows were implanted with subcutaneous PIT tags (Micro Products Australia Mini Microchips, 1.4 × 8.5 mm, 0.52 g) and were recorded when they entered and when they exited the feeding chamber. PIT tags were read approximately every 0.5 s.
3. Great tit in Wytham woods, Oxfordshire, UK (51.46 N, 01.20 W): We used data collected between December 2011 and February 2012 from eight feeders at the northern extent (the Great Woods) of Wytham Woods, a largely broadleaf deciduous woodland surrounded by open arable farmland. Pairs of great tits establish territories during the breeding season, but this territoriality breaks down during the autumn and winter, when birds form loose fission-fusion flocks with unrelated individuals that forage together (Hinde 1952). Bird feeders had two access ports, both fitted with an RFID antenna (Dorset ID, Netherlands, Fig. 1Bc). The feeders were operational every week, from pre-dawn Saturday morning until after dusk on Sunday evening, and PIT tags were read approximately every 0.25 s.
4. Sociable weavers in Benfontein Nature reserve, Northern Cape, South Africa (28.51 S, 2.46E): Sociable weavers are highly social, colonial nesting passerines, endemic to Southern Africa. Outside of the breeding period they form large gregarious foraging flocks composed of mixed family groups (Ferreira et al. 2020). We collected social data at two feeder boxes placed next to each other on the ground (Fig. 1Bd) during the breeding season, between December 2017 and February 2018. Each feeder box had four perches, each with an attached RFID antenna (Priority 1 RFID, Melbourne, Australia), over four feeding trays. Although this setup only allowed one bird to access a feeding tray at once, four birds could feed at adjacent perches at the same time, and these co-occurring birds were considered as feeding at the same time during network construction. All captured weavers were fitted with a PIT tag mounted to a plastic leg-ring. PIT tags were read approximately every 0.5 s.

### Social network construction using different association definitions

We built three networks for each system, one for each association definition (Figs. 1), (1) time-window (Fig. 1Aa); (2) GMM (Psorakis et al. 2012, 2015; Farine 2017; Fig. 1Ab); (3) arrival-time definitions (Dunning et al. 2023; Fig. 1Ac). We built weighted networks, scaled using the simple ratio index (Farine and Whitehead 2015), and undirected networks in R (R Core Team 2023). We applied these general parameters between systems:

1. Strict time-window (Fig. 1Aa): The time-window approach had a single overlap parameter (**Δ**t), where two individuals who visited a feeder within **Δ**t were defined as associates. In the current study, we hope to compare other methods with the strictest definition for association, so we defined **Δ**t as one second to capture absolute physical and temporal proximity at the feeder (for example, Farine 2015; McCully and Rose 2023).
2. GMM (Fig. 1Ab): We used the GMM function in the asnipe R package (Farine 2013) to detect groups. The GMM function detects the start and end point of gathering events and associates all individuals (Psorakis et al. 2012, 2015). We combined the date and location parameters within each system into a unique location to reduce processing time (Farine 2017A).
3. Arrival-time (Fig. 1Ac): We built arrival networks using a series of custom R functions, (see Chan and Dunning 2023). This method assumes that birds arriving together are associated before they arrive at the resource, and therefore seeks to detect arrival time by which to define associations. We therefore defined two parameters: (1) A time threshold within which two (or more) individuals are associated based on their arrival times (**Δ**t). (2) A period of inactivity for each bird, after which a bird is considered to have left the feeder (**Δ**i), allowing for a new arrival. We defined **Δ**t as 150 s and **Δ**i as 300 s following Dunning et al. (2023), based on the biology of house sparrows on Lundy Island. The definition of these thresholds may alter network structure, and so we compared variations of **Δ**t in sensitivity analyses (see Sensitivity Analysis).

For both strict time-window and arrival-time definitions, we employed a chain-rule method to assign individuals into groups, based on the gambit of the group principle. For example, under both time-window and arrival-time definitions, if individuals A and B visit a feeder within t of each other, then immediately C visits within t of B, but not A, they are connected into a single group (A-B, B-C and C-B). Whereas, the arrival time definition introduces a parameter to prevent long chains of connection, by measuring association only from the point of arrival; if A and B arrive within t of each other, then after an interval long enough to determine that they have likely left the feeding area, B and C arrived within t of each other but without A, we instead infer two groups (A-B and B-C). If C arrives alone, even if A and B are still present but arrived much earlier, it is not linked to either of the others; see Fig. 1).

We used the iGraph package in R (Csardi and Nepusz 2006) to extract three node-based network measures from the association datasets for each of the four systems: degree, the number of unique associates connected to a focal individual; strength, the total number of associations between a focal individual and all associates; and weighted betweenness, the number of geodesics (shortest paths between any nodes) that pass through a focal individual. For weighted betweenness, we additionally inversed the network weights as igraph considers network weights as costs (Silk et al. 2017). Individuals who had a degree of 0 were removed from the network.

### Analysis 

- Similarity in network structures
  We described the cardinality of for each network for each association definition, i.e. the number of individuals (Vertices; V), and the number of associations (Edges, E), as well as the network density (D). The density of a network is defined by the number of observed edges over the maximum potential edges. Then, we used two Jaccard similarity indices to compare global network structures between all possible pairs of association definitions, within systems using the multinet R package (Magnani et al. 2021). Following (Bródka et al. 2018; but see Emmert-Streib et al. 2016): (1) Jaccard edges to compare common edges; and (2) Jaccard triangles as a measure of common clusters of individuals between networks. All Jaccard similarities range between 0 and 1, where 0 denotes no overlap between networks, and 1 when networks are identical.
  Finally, we ran pairs of Multiple Regression Quadratic Assignment Procedure (MRQAP) network regression using the ‘netlm’ function in the ‘sna’ R package (Butts 2016; Elmer 2021), that allowed for the relationship between two networks to be quantified, including edge weights. We first extracted and scaled the weighted adjacency matrices for each network, then ran a simple regression to determine whether one network predicts the other. The extracted effect size can then determine the extent in which a pair of networks were similar. In addition, we also ran Mantel tests (Mantel 1967; Croft et al. 2008) using the Pearson correlation method and 999 matrix permutations with the ‘Vegan’ R package (Dixon 2003). Theis method determines significance by randomizing one of the two matrices and extracting an expected null distribution, where p-value represents the proportion of the null distribution more extreme than the actual estimate. The results produce a correlation between networks that varies between − 1 and 1, representing matrices being negatively and positively correlated respectively.
  For each jaccard similarity index, MRQAP regression and mantel test described above, we compared metrics obtained from observed networks generated with 1000 permuted networks yielding a distribution of social parameters that would be expected if individuals were in random groups at the feeder. We adopted a pre-network permutation approach (Farine 2017), by first extracting the individual group occurrence matrix (or “group-by-individual” matrix in asnipe) using each association definition, which describes each detected group and its members (marked as a 1 or 0). We then shuffled each individual’s occurrence column without replacement to ensure that each individual was detected in the same number of groups at the feeder, but group membership was randomised. We repeated the permutation for each dataset, i.e. for each species and association definition, then extracted the same jaccard similarity, MRQAP and mantel coefficients. Finally, we calculated the proportion of data in the randomised distribution that is more extreme than the observed estimate.
- Repeatability of social traits
  We constructed weekly sub-graphs across each recording period for each of our four avian systems, using each of the three definitions for *association*. We extracted three network measures from each subgraph for each week, then z-transformed to normalise the measures due to differences in network structure across weeks.
  Individual social network metrics are an aspect of an individual’s animal personality and have been shown to be consistent across various study systems (Bell et al. 2009; Aplin et al. 2015; Hillemann et al. 2019; Tkaczynski et al. 2020; Proops et al. 2021; Ogino et al. 2023). Thus, in the four study systems tested here, we assume that if individuals do display consistent differences in social network position, then an appropriate definition of association should find repeatability of social traits over time.
  We analysed repeatability of social traits using the R package MCMCglmm (Hadfield 2010), using package default function hyper-parameters of 13,000 iterations, thinning interval of 10 and burn-in of 3,000 iterations. We modelled each social trait as a response variable against the model intercept and with individual ID as a random effect. Repeatability was defined as the variance explained by individual ID over the total variance (Nakagawa and Schielzeth 2010). We describe the repeatability in the context of three levels, low (< 0.3), medium (0.3–0.5) and high (> 0.5; following Bell et al. 2009; Winney et al. 2018). Using these subgraphs, we also explored the relationship between individual network measures extracted using each association definition, by running linear regressions for each association definition pair for all bird individuals across all weeks.
- Sensitivity Analyses
  Finally, to test for how parameterisation of the arrival time and strict time-window method affects resulting networks, we computed repeatability measures again but varying the **Δ**t parameter from 1 to 300 s for both the arrival and strict time-window methods. For the arrival time approach, we maintained **Δ**i at 300 s. We did not do this for GMM networks, since associations defined by GMM automatically set parameters within the detection algorithm.

## Results

We built social networks using 286,669 RFID detections comprising of 118 individuals visiting feeders on Lundy Island, 27,456 detections of 66 individuals at Broken Hill, 402,255 detections of 219 individuals at Wytham Woods and 197,857 detections of 62 individuals at Benfontein. The number of individuals (network nodes) and association (edges) and their centrality varied with association definition (Table 1). 

**Fig. 2:**
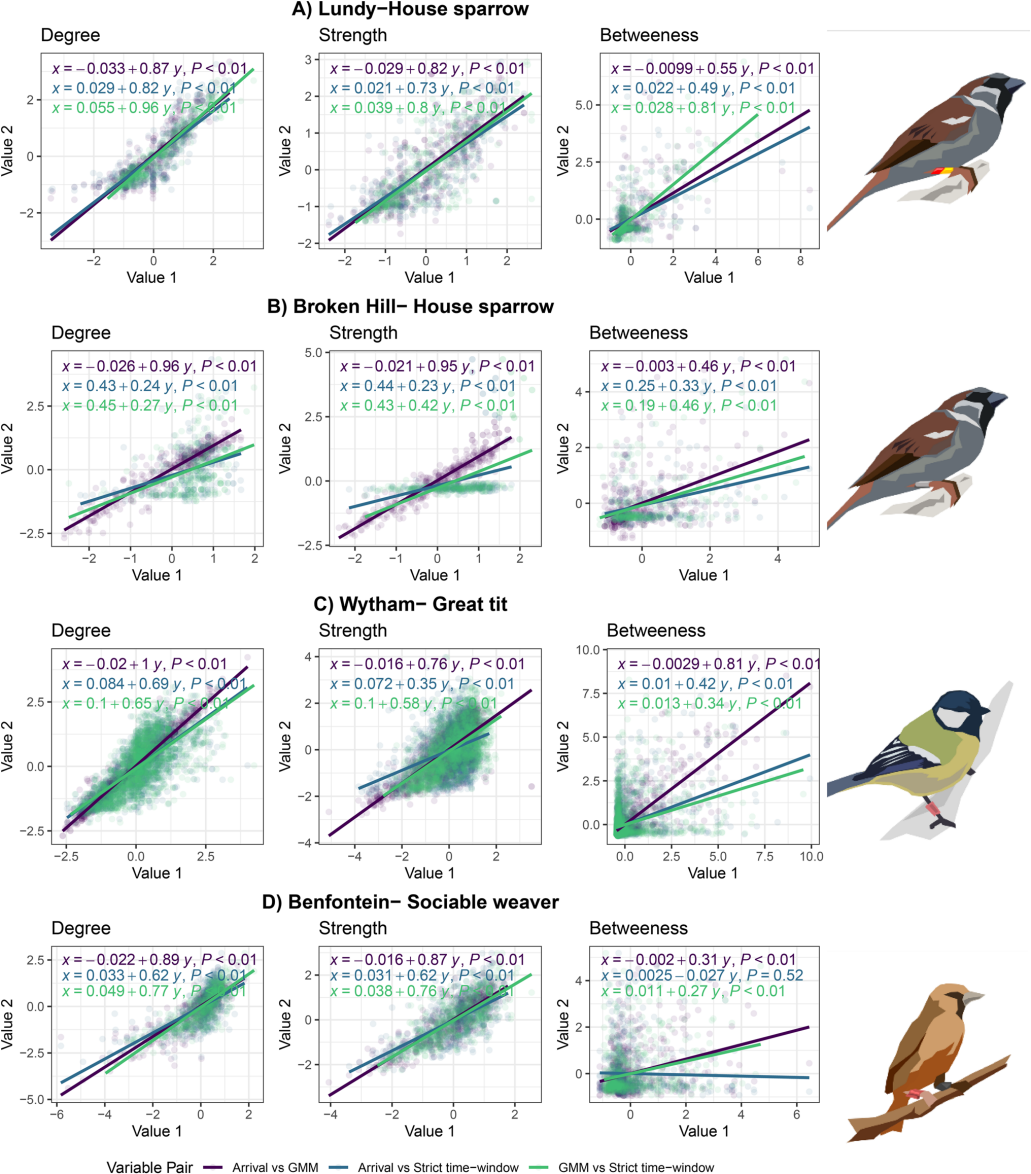
Linear regression results of three individual social network traits (z-transformed within weeks) extracted from weekly networks from four study systems using three association definitions. **A**) House sparrow data in Lundy Island, UK; **B**) House sparrow data in Broken Hill, Australia; **C**) Great tits data in Wytham Woods, UK; **D**) Sociable Weaver data in Benfontein Nature Reserve, South Africa. We ran linear regressions for value 1 ~ value 2, reported as value 1 vs. value 2: Arrival time vs. GMM (Purple), Arrival time vs. Strict time-window (Blue) and GMM vs. Strict time-window (Green), with the value 1 on the x-axis and value 2 on the y-axis. P-values represents significance of model estimate
  Using Jaccard similarity indices and MRQAP regressions to compare similarities between networks, we show that network structures are robust to changing association definition (Table 1). All extracted similarity metrics were significant when compared with a null distribution of random networks (Supplementary Tables 1, 2). We also report Mantel test results in Supplementary Table 3, though the results are similar to MRQAP regressions.
  From the Jaccard similarity metrics, the metrics are generally high, with the number of individuals detected (vertices; cardinality V) being similar between network pairs, whereas metrics to capture clustering (Jaccard triangles; 0.01–0.77), dyad identity (Edges, cardinality E; 0.15–0.87) and associated edge weights (MRQAP regression coefficient; 0.46–0.92) varied more. We found the highest similarity scores between the GMM and arrival-time definitions, followed by GMM and strict time-window definitions, then strict time-window and arrival definitions. All definitions included a similar number of individuals in networks, but the number of edges differed. Specifically, networks based on strict time-windows produced less dense networks, while using the arrival-time definition resulted in denser networks, likely due to the large difference in threshold used between the two methods.
- Repeatability of social traits
  We calculated repeatability over nine weeks at Broken Hill, Australia, fourteen weeks on Lundy Island (see Dunning et al. 2023), thirteen weeks at Benfontein Nature Reserve, South Africa; and fourteen weeks at Wytham woods (see Aplin et al. 2015a). We found that degree and strength, are repeatable in all four systems, with little variation between association definitions (Fig. 3). Repeatability was lower for betweenness in all systems, but still largely similar between association definitions. Between study systems, repeatability was low for all three definitions in the Lundy systems; low-moderate repeatability in the Wytham system; and high repeatability for degree and strength, but low – moderate repeatability for betweenness in Broken Hill and Benfontein systems. Repeatability of degree and strength was significantly lower in the Wytham system when associations were defined by the strict time-window definition (Fig. 3C). The arrival-time and GMM definitions performed equally across systems (Fig. 3).

- Similarity in network structures
  Using the network centrality metrics extracted from each weekly sub-graph, we show that degree and strength positively predicts each other across all systems (regression coefficients ~ 0.6 – ~0.9; Fig. 2) with lower coefficients for betweenness (~ 0.3 – ~0.7)). Other than the house sparrows on Lundy Island (Fig. 2A), the regression coefficient between arrival and GMM were always highest for all traits.

**Fig. 3:**
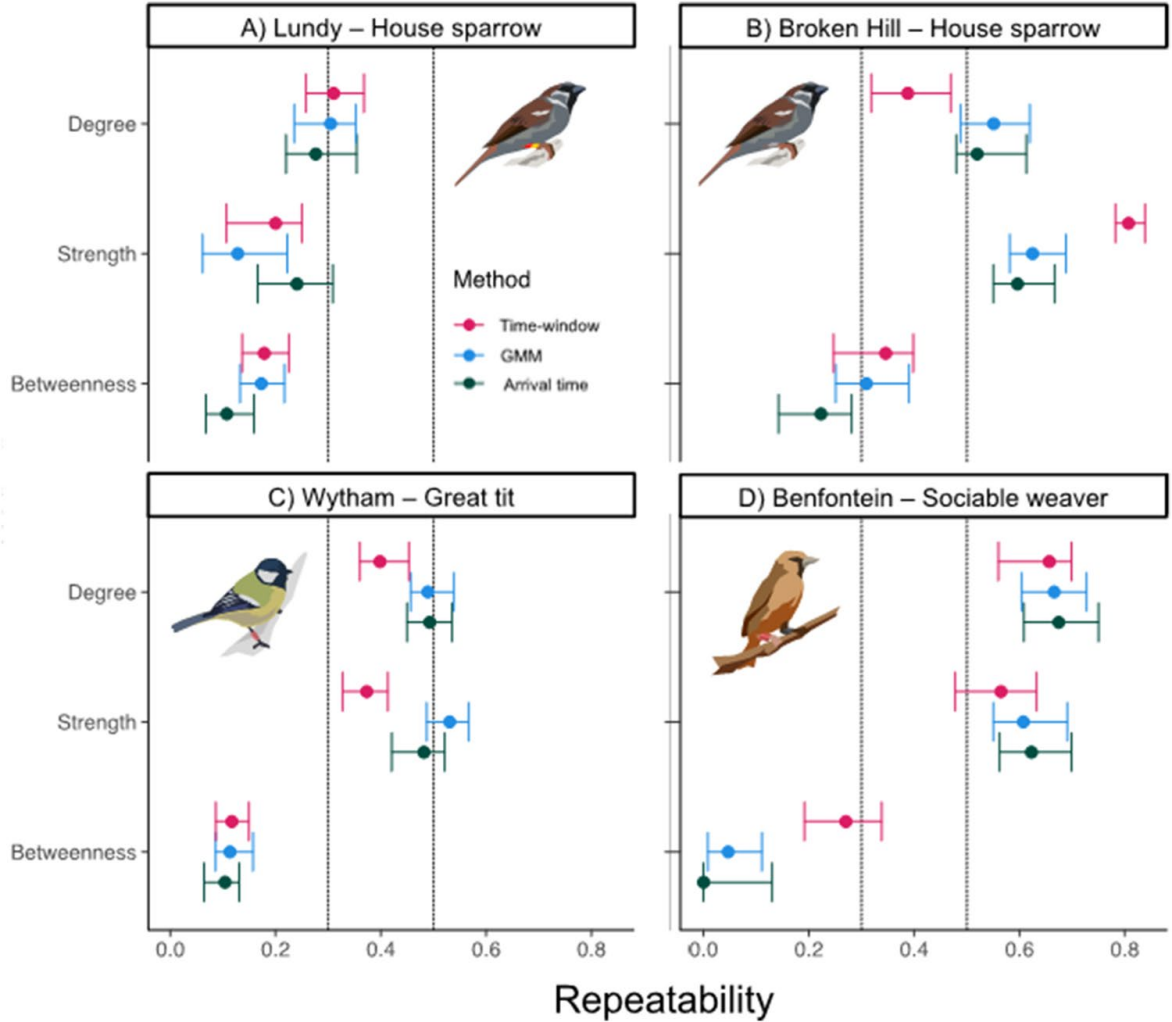
Between week repeatability for three individual social traits (degree, strength and betweenness), between three association definitions and in four systems: **A**) House sparrows from Lundy Island, UK, **B**) House sparrow data from Broken Hill, Australia; **C**) Great tits data from Wytham Woods, UK; and **D**) Sociable Weaver data from Benfontein Nature Reserve, South Africa. Points denote the mean repeatability, and error bars the 95% CIs. Dotted lines denote thresholds for low (< 0.3), medium (0.3 - 0.5) and high (> 0.5) repeatabilities (also see Table 2)
- Sensitivity Analyses
  Finally, we explored how altering the time parameter (**Δ**t) affected network structure within the arrival-time and strict time-window methods. For the arrival-time definition, our results show that social traits have low repeatability with a low **Δ**t, but increased as **Δ**t increases until reaching a plateau. We report a similar plateau for the time-window method, without an initial increase in repeatability. The repeatability of betweenness was more sensitive to changes for both methods, especially in Broken Hill and Lundy Island (Fig. 4).

**Fig. 4:**
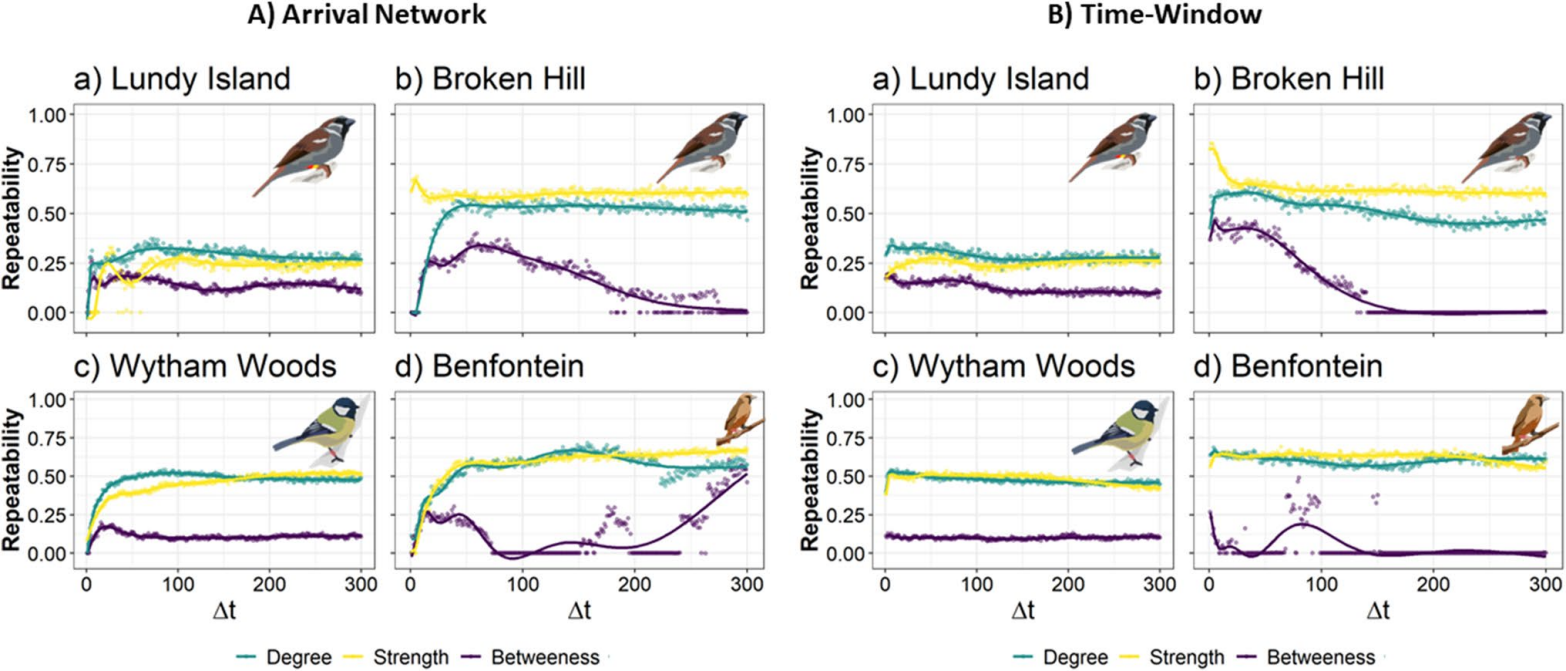
Sensitivity analysis of between week repeatability of individual metrics for arrival and time-window networks. For arrival time networks, we vary Δt from 0 to 300 seconds, and fixed Δi = 300s, and for time-window networks we varied Δt from 0 to 300 seconds. For associations defined by **A**) arrival time and **B**) time window, across four systems: **a**) House sparrow data in Lundy Island, UK; **b**) House sparrow data in Broken Hill, Australia;** c**) Great tits data in Wytham Woods, UK; **d**) Sociable Weaver data in Benfontein Nature Reserve, South Africa. Repeatability was calculated for degree (number of edges per node; green), strength (weighted degree; yellow) and betweenness (number of shortest paths passing through a node; purple)

**Table 1:** We compared three network structures, built using three association definitions, for four systems: (A) House sparrow on Lundy Island, UK. (B) House sparrow in Broken Hill, Australia (C) Great tits in Wytham Woods, UK: (D) Sociable Weaver in Benfontein Nature Reserve, South Africa. (1) we report the cardinality of each network in the first row as vertices (V; individuals in each network), edges (E; associations between individuals) and network density (D; the number of edges over the total number of potential edges); (2) we described two Jaccard similarity measures between networks (lower left matrixA-D): edges (common dyadic edges); triangles (common clusters of triads). Finally, we report the effect size from a MRQAP regression (upper right matrix A-D), which quantifies how well one network predicts the other. All comparison metrics are significant (*p* < 0.05) when compared to a null distribution generated by pre-network permutation, hence is not reported here

Definitions		**A. Lundy**			**B. Broken Hill**		
		Strict-time window	GMM	Arrival time	Strict-time window	GMM	Arrival time
Cardinality (V/D/E)		118/1619/0.23	118/2274/0.32	119/3783/0.54	57/290/0.18	66/1862/0.73	66/1534/0.71
Strict-time window	-	-	0.73	0.46	-	0.31	0.27
GMM	Edges Triangles	0.7 0.5	-	0.63	0.15 0.01	-	0.86
Arrival time	Edges Triangles	0.42 0.16	0.58 0.32	-	0.18 0.02	0.82 0.67	-
System		**C. Wytham**			**D. Benfontein**		
Cardinality (V/E/D)		204/4115/0.29	219/6774/0.28	214/6688/0.19	62/978/0.51	62/1433/0.75	62/1583/0.83
Strict-time window	-	-	0.75	0.72	-	0.58	0.43
GMM	Edges Triangles	0.6 0.37	-	0.94	0.68 0.49	-	0.85
Arrival time	Edges Triangles	0.61 0.38	0.94 0.91	-	0.61 0.4	0.87 0.77	-

**Table 2:** Repeatability and mean measures for three social network traits (degree, strength, betweenness) across weeks in 4 study systems (Lundy Island House sparrows, Broken Hill House Sparrows, Wytham Woods Great tits, and Benfontein Sociable Weavers) using three association definitions. We calculated for each metric across all weeks within study systems, with standard deviation provided in parenthesis

System	Trait	Method	Mean (SD)	*R*	95% CI	System	Mean (SD)	*R*	95% CI
Lundy	Degree	Strict-time window	13.38 (16.08)	0.3	(0.26–0.37)	Broken Hill	3.41 (2.8)	0.39	(0.32–0.48)
		GMM	18.63 (20.61)	0.31	(0.26–0.38)		26.38 (13.94)	0.58	(0.49–0.62)
		Arrival	32.33 (31.93)	0.28	(0.23–0.35)		25.01 (13.93)	0.55	(0.47–0.61)
	Strength	Strict-time window	0.12 (0.13)	0.17	(0.1–0.25)		0.09 (0.24)	0.81	(0.78–0.84)
		GMM	0.78 (0.77)	0.21	(0.14–0.3)		1.71 (0.89)	0.64	(0.58–0.69)
		Arrival	0.28 (0.19)	0.22	(0.15–0.29)		0.38 (0.17)	0.61	(0.55–0.67)
	Betweenness	Strict-time window	44.33 (123.2)	0.18	(0.11–0.24)		25.22 (64.08)	0.16	(0.02–0.23)
		GMM	37.2 (104.76)	0.00	(0.00–0.16)		19.49 (29.75)	0.11	(0.16–0.31)
		Arrival	24.81 (72.27)	0.07	(0.00–0.14)		17.61 (22.87)	0.22	(0.16–0.31)
Wytham Woods	Degree	Strict-time window	12.84 (7.8)	0.4	(0.36–0.45)	Benfontein	18.36 (10.11)	0.61	(0.55–0.7)
		GMM	30.41 (14.51)	0.5	(0.46–0.54)		30.02 (11.26)	0.67	(0.59–0.73)
		Arrival	29.26 (14.35)	0.5	(0.45–0.54)		34.74 (10.67)	0.68	(0.6–0.74)
	Strength	Strict-time window	0.1 (0.05)	0.37	(0.32–0.41)		0.19 (0.11)	0.53	(0.45–0.63)
		GMM	4.15 (1.96)	0.52	(0.48–0.56)		2.12 (0.95)	0.62	(0.54–0.69)
		Arrival	0.45 (0.13)	0.46	(0.41–0.51)		0.44 (0.14)	0.63	(0.56–0.7)
	Betweenness	Strict-time window	135.8 (303.26)	0.09	(0.06–0.12)		26.86 (45.42)	0.19	(0.12–0.27)
		GMM	140.48 (385.07)	0.08	(0.06–0.12)		19.37 (26.73)	0.11	(0.05–0.18)
		Arrival	134.11 (390.52)	0.13	(0.09–0.17)		14.78 (32.52)	0.05	(0.01–0.11)

## Discussion

In this study, we compared three association definitions applied to edges in avian social networks in four study systems. To define an association, we used a strict time-window approach (Farine 2015), gaussian mixture models (GMM; Psorakis et al. 2012, 2015), and arrival-time (Chan and Dunning 2023; Dunning et al. 2023). We find that different association definitions yielded comparable individual-level social traits. When constructing animal social networks, the association definition should fit the research question, the social system of the study species, and other characteristics of the study design, such as technical limitations (Croft et al. 2008; Farine and Whitehead 2015).

We found statistically significant similarity of social indices within systems, high level of inter-dependence between individual centrality traits, and similar levels of repeatability between association definitions. We found that some association definitions resulted in more similar networks between systems with more similar ecologies, for example in the highly gregarious and open-access antenna systems at Lundy and Benfontein. Altering the arrival-time overlap (**Δ**t) within arrival and time-window networks was also generally robust to the definition of **Δ**t across systems. Association definitions generally did not significantly change network size, but using the strict time-window at Broken Hill led to fewer individuals being included in the network. Overall, processing of the same temporal data-stream using the three association definition methods applied here resulted in similar social networks. However, we show that methodological decisions can result in subtle differences, mainly explained by (1) differences between association definitions in the context of the system ecology, and (2) RFID feeder design.

We found slight differences in social networks created based on the association definition used across all four study systems. Particularly, the GMM and arrival method generally produced more similar networks across the four study systems compared to strict-time window, evident from the higher similarity metrics and consistent repeatability of social traits across methods. The strict-time window method uses a low (one second) threshold to identify associations, resulting in closer co-occurrence to be treated as an association, in contrast to groups identified using the GMM or arrival approach. This distinction is important for the research question at hand, as researchers interested in capturing closer associations might opt for a strict-time window method, whereas the GMM or arrival methods might be more appropriate for research questions related to capturing wider group compositions. However, in the house sparrow system on Lundy Island, the strict-time window and GMM methods produced more similar networks compared to the arrival network. This may be explained by the gregarious nature of house sparrows that often aggregate at feeders in large numbers, making the arrival approach capture subtly different associations that are based on the behaviour outside of a feeder instead of proximity in the feeder.

Next, our results also suggest that the design of RFID feeders can influence the obtained social networks using different association methods. When using the strict time-window definition, we observed lower repeatability of individual social traits in Wytham woods, but increased repeatability in Broken Hill and Benfontein. Importantly, RFID feeders in Wytham Woods only allow up to two birds to feed simultaneously, and since we used a strict threshold of 1 s to define the time-window, this method seems to have identified less repeatable associations compared to, for example, the GMM approach. Similarly, the strict time-window definition in Broken Hill resulted in smaller network sizes, since sparrows were only detected as they entered and left a feeding chamber, affecting the detection of groups. In the Broken Hill sparrow system, a higher **Δ**t value (e.g. 5–10 s) might be more appropriate to capture physical and temporal proximity. On the other hand, shorter time-windows can be more appropriate for open RFID systems like in Benfontein and Lundy Island (Fig. 1A), where multiple individuals can access the food resource at one time. Overall, GMM and arrival definitions captured more similar networks compared to strict time-window approaches, because the latter is capturing physical and temporal proximity, while the two former capturing larger foraging groups.

The sensitivity analysis suggested that both the arrival time and strict time window approaches are robust against changes in the time-window parameter. However, betweenness seemed to be much more variable, especially in the house sparrow systems on Lundy Island and Broken Hill. This was likely due to the sensitivity of the betweenness measure relative to the resulting network size and fine-scaled topological differences within a network. Where degree and strength captures the variation in each individual’s primary social associates, betweenness (the number of shortest paths a node sits), is more sensitive to the changing structure of the network. It may also be the case where RFID data of house sparrows contains more noise from random aggregations at the feeder, which makes the definition of Δt more important in the resulting network.

When deciding which association definition to use, researchers should not only consider which associations definition is appropriate for the research question at hand, but also consider the effect of choosing different time-window parameters when constructing social networks. We provide code for a comparison between association definitions and sensitivity analysis, and we encourage researchers to explore how different definitions affect the resulting networks, and make methodological decisions considering the biology of their study species.

A core limitation of our study (and indeed, of many studies on social behaviour in the wild) is the inability to know whether the association definition we use are capturing any social relationship or preferences between individuals. While RFID readers at feeders is one of the most common ways to measure social association in passerine bird studies, the method does not measure specific behavioural interactions (in contrast to, for example, primate social network that are based on observed grooming interactions) and can also lead to artificially created associations. In other words, these remotely-sensed networks are only a proxy of the underlying social structure in a population, and whether this social structure is appropriate will depend on the research question at hand.

While the research question should be the primary consideration when deciding on the association definition of choice, we introduce additional considerations that can influence the constructed social networks when paired with a specific association definition. Key considerations are: (1) The gregariousness of the study system, or their propensity to aggregate at a resource (Krause and Ruxton 2002), and (2) Data recording methodology; for example, use of open-access systems where multiple individuals can feed and get recorded simultaneously versus systems which limited access to the feeder and antenna. Where the concepts of social association are unclear, it may be beneficial to compare different definitions before hypothesis testing. Furthermore, we encourage researchers to report justification of association definition in published research, providing a biological justification on the choice when testing hypotheses using animal social networks. These could be empirically tested against another variable like familial ties (e.g. Ferreria 2020) or based on observation of the study species (e.g. Dunning et al. 2023). While we show here that differing association definitions are generally robust and produce broadly similar networks, any decisions researchers make nevertheless result in different network structures and may influence research outcomes.

## Supplementary Information

Below is the link to the electronic supplementary material.

ESM1 (DOCX 16.8 KB)

## Data Availability

All code scripts used to generate arrival networks are available on the Zenodo repository (Chan and Dunning 2023: 10.5281/zenodo.7527440). All data and code that can be used to reproduce results are available here: 10.5281/zenodo.7892571.
