# Supplementary material for: Animal social networks are robust to changing association definitions
Source: Behav Ecol Sociobiol. 2025 Feb 6;79(2):26. doi: 10.1007/s00265-025-03559-7 (PMC11802709; doi:10.1007/s00265-025-03559-7)
Supplement: Supplementary file 1 — (DOCX 16.8 KB) [file 265_2025_3559_MOESM1_ESM.docx]

Animal Social Networks are Robust to Changing Association Definitions.

*Alex Hoi Hang Chan^1,2,3^, *Jamie Dunning^3,4^, Kristina B Beck^5^, Terry Burke^6^, Heung Ying Janet Chik^7,8^, Daniel Dunleavy^3^, Tim Evans^9^, André Ferreira^10^, Babette Fourie^10,11^, Simon C. Griffith^8^, Friederike Hillemann^12^ and Julia Schroeder^3^.

*Shared first authorship

Corresponding author –  [jd2819@ic.ac.uk](mailto:jd2819@ic.ac.uk)

Supplementary Table 1

Null distributions for all network comparison metrics used in the study. 1000 permutations were done for each of the 5 comparison metrics using 3 association methods across 4 study systems. For each permutation, two random networks were created, and each similarity metric were computed to create a randomized distribution. We present the mean value of the random distribution and upper and lower 95% higher density intervals.

|  | Strict time-window | | GMM | | Arrival time | |
| --- | --- | --- | --- | --- | --- | --- |
|  | Mean | 95% hdi | Mean | 95% hdi | Mean | 95% hdi |
| a) Lundy Island | | | | | | |
| Jaccard Edges | 0.55 | 0.54-0.57 | 0.55 | 0.54-0.57 | 0.47 | 0.45-0.49 |
| Jaccard Triangles | 0.44 | 0.42-0.46 | 0.45 | 0.43-0.47 | 0.37 | 0.35-0.39 |
| MRQAP coefficient | 0.29 | 0.25-0.32 | 0.30 | 0.27-0.34 | 0.23 | 0.19-0.25 |
| MRQAP intercept | -0.02 | -0.03--0.01 | -0.02 | -0.03--0.01 | -0.01 | -0.02--0.003 |
| Mantel coefficient | 0.79 | 0.74-0.84 | 0.73 | 0.64-0.79 | 0.44 | 0.31-0.53 |
| b) Broken Hill | | | | | | |
| Jaccard Edges | 0.73 | 0.72-0.75 | 0.79 | 0.77-0.80 | 0.73 | 0.72-0.75 |
| Jaccard Triangles | 0.63 | 0.60-0.65 | 0.69 | 0.66-0.71 | 0.61 | 0.59-0.64 |
| MRQAP coefficient | 0.44 | 0.40-0.48 | 0.47 | 0.43-0.51 | 0.39 | 0.36-0.43 |
| MRQAP intercept | -0.02 | -0.04--0.01 | -0.03 | -0.04--0.012 | -0.03 | -0.05--0.02 |
| Mantel coefficient | 0.80 | 0.78-0.83 | 0.81 | 0.79-0.82 | 0.71 | 0.68-0.73 |
| c) Wytham woods | | | | | | |
| Jaccard Edges | 0.68 | 0.67-0.70 | 0.79 | 0.79-0.80 | 0.70 | 0.69-0.71 |
| Jaccard Triangles | 0.57 | 0.56-0.59 | 0.72 | 0.71-0.72 | 0.60 | 0.58-0.62 |
| MRQAP coefficient | 0.50 | 0.46-0.54 | 0.53 | 0.52-0.55 | 0.52 | 0.49-0.55 |
| MRQAP intercept | -0.01 | -0.01--0.003 | 0.001 | -0.003-0.006 | -0.007 | -0.01--0.004 |
| Mantel coefficient | 0.87 | 0.84-0.89 | 0.86 | 0.85-0.87 | 0.90 | 0.83-0.92 |
| d) Benfontein | | | | | | |
| Jaccard Edges | 0.86 | 0.85-0.88 | 0.83 | 0.81-0.85 | 0.83 | 0.82-0.85 |
| Jaccard Triangles | 0.81 | 0.79-0.83 | 0.76 | 0.74-0.78 | 0.76 | 0.74-0.78 |
| MRQAP coefficient | 0.75 | 0.70-0.80 | 0.70 | 0.66-0.75 | 0.67 | 0.64-0.71 |
| MRQAP intercept | -0.04 | -0.07- -0.02 | -0.05 | -0.07--0.03 | -0.05 | -0.07- -0.03 |
| Mantel coefficient | 0.96 | 0.96-0.97 | 0.93 | 0.92-0.93 | 0.89 | 0.88-0.90 |

Supplementary Table 2

MRQAP network regression estimates and p-values. We built networks using 3 different association networks across 4 study systems, and compared each unique pair of networks using and MRQAP regression. We report the coefficient estimate of the network and the intercept. P-values were calculated by the proportion of data from the null distribution that is extreme than the actual value.

|  | Variable | Estimate | p-value |
| --- | --- | --- | --- |
| A) Lundy | | | |
| GMM/ Arrival time | Coefficient | 0.63 | 0 |
|  | Intercept | -0.01 | 0.52 |
| Arrival time / Strict time window | Coefficient | 0.46 | 0 |
|  | Intercept | -0.01 | 0.51 |
| Strict time window / GMM | Coefficient | 0.73 | 0 |
|  | Intercept | -0.01 | 0.51 |
| B) Broken Hill | | | |
| GMM/ Arrival time | Coefficient | 0.86 | 0 |
|  | Intercept | -0.01 | 0.50 |
| Arrival time / Strict time window | Coefficient | 0.27 | 0 |
|  | Intercept | -0.03 | 0.48 |
| Strict time window / GMM | Coefficient | 0.31 | 0 |
|  | Intercept | -0.02 | 0.50 |
| C) Wytham | | | |
| GMM/ Arrival time | Coefficient | 0.94 | 0 |
|  | Intercept | 0.00 | 0.48 |
| Arrival time / Strict time window | Coefficient | 0.72 | 0 |
|  | Intercept | -0.01 | 0.50 |
| Strict time window / GMM | Coefficient | 0.75 | 0 |
|  | Intercept | 0.00 | 0.48 |
| D) Benfontein | | | |
| GMM/ Arrival time | Coefficient | 0.85 | 0 |
|  | Intercept | -0.03 | 0.49 |
| Arrival time / Strict time window | Coefficient | 0.43 | 0 |
|  | Intercept | -0.10 | 0.46 |
| Strict time window / GMM | Coefficient | 0.58 | 0 |
|  | Intercept | -0.08 | 0.49 |

Supplementary Table 3

Mantel tests correlating each unique pair of 3 association definition across 4 study systems. Mantel coefficients range from -1 to 1, describing whether two networks are correlated or not, while null confidence intervals and p-values were obtained from 999 matrix permutations.

|  | Variable | Estimate |
| --- | --- | --- |
| A) Lundy | | |
| GMM/ Arrival time | Mantel coefficient | 0.75 |
|  | p-value | 0.001 |
|  | Null confidence interval | -0.06 - 0.06 |
| Arrival time / Strict time window | Mantel coefficient | 0.54 |
|  | p-value | 0.001 |
|  | Null confidence interval | -0.05 – 0.06 |
| Strict time window / GMM | Mantel coefficient | 0.81 |
|  | p-value | 0.001 |
|  | Null confidence interval | -0.04-0.04 |
| B) Broken Hill | | |
| GMM/ Arrival time | Mantel coefficient | 0.92 |
|  | p-value | 0.001 |
|  | Null confidence interval | -0.09 – 0.1 |
| Arrival time / Strict time window | Mantel coefficient | 0.46 |
|  | p-value | 0.001 |
|  | Null confidence interval | -0.02 – 0.05 |
| Strict time window / GMM | Mantel coefficient | 0.69 |
|  | p-value | 0.001 |
|  | Null confidence interval | -0.02 – 0.04 |
| C) Wytham | | |
| GMM/ Arrival time | Mantel coefficient | 0.94 |
|  | p-value | 0.001 |
|  | Null confidence interval | -0.02 – 0.02 |
| Arrival time / Strict time window | Mantel coefficient | 0.74 |
|  | p-value | 0.001 |
|  | Null confidence interval | -0.02 – 0.02 |
| Strict time window / GMM | Mantel coefficient | 0.82 |
|  | p-value | 0.001 |
|  | Null confidence interval | -0.02 – 0.02 |
| D) Benfontein | | |
| GMM/ Arrival time | Mantel coefficient | 0.92 |
|  | p-value | 0.001 |
|  | Null confidence interval | -0.02 – 0.02 |
| Arrival time / Strict time window | Mantel coefficient | 0.47 |
|  | p-value | 0.001 |
|  | Null confidence interval | -0.1- 0.1 |
| Strict time window / GMM | Mantel coefficient | 0.64 |
|  | p-value | 0.001 |
|  | Null confidence interval | -0.02 – 0.02 |
